# Miller Fisher Syndrome With Syndrome of Inappropriate Antidiuretic Hormone Secretion: A Case Report

**DOI:** 10.7759/cureus.83981

**Published:** 2025-05-12

**Authors:** Khaing Zin That, Kai Xiong Lim, Marc Hai Liang Wong

**Affiliations:** 1 Internal Medicine, Sengkang General Hospital, Singapore, SGP

**Keywords:** anti-gq1b, hyponatremia, miller fisher syndrome (mfs), opthalmoplegia, syndrome of inappropriate secretion of antidiuretic hormone (siadh)

## Abstract

We report the case of a 70-year-old man with an incomplete form of Miller Fisher syndrome (MFS) complicated by syndrome of inappropriate secretion of antidiuretic hormone (SIADH). He presented with diplopia two weeks after a respiratory infection. Neurological examination showed bilateral pupil-sparing third nerve palsy, with normal reflexes, gait, coordination, power, and sensation. Serum sodium was markedly low (112 mmol/L) at presentation, with low serum osmolality, high urine sodium, and elevated urine osmolality. Anti-GQ1b IgG antibodies were positive, confirming the diagnosis of MFS. He was managed supportively with fluid restriction and oral sodium chloride tablets. He did not receive intravenous immunoglobulin. Symptoms resolved completely after eight weeks. This case underscores the importance of recognizing incomplete presentations of MFS and highlights the rare but clinically significant complication of SIADH. Early identification and appropriate management of both conditions are critical to improving patient outcomes and preventing complications.

## Introduction

Miller Fisher syndrome (MFS) was first described in 1956 as a variant of Guillain-Barré syndrome (GBS) [1], characterized by acute neuropathy from inflammation mediated by antibodies against neuronal membrane gangliosides. MFS is strongly associated with anti-GQ1b IgG antibodies, where GQ1b ganglioside is abundantly present in cranial nerves III, IV, and VI and the dorsal root ganglia [2,3]. The classical presentation of MFS is a triad of ophthalmoplegia, ataxia, and areflexia. However, the syndrome may not always manifest fully, resulting in diagnostic confusion. Incomplete forms, such as isolated ophthalmoplegia without ataxia or areflexia, can lead to delayed or missed diagnoses [4,5]. While hyponatremia secondary to the syndrome of inappropriate secretion of antidiuretic hormone (SIADH) is well-documented in GBS, its occurrence in MFS is considerably less common and infrequently reported.

## Case presentation

A 70-year-old male with a history of hypertension was brought to the emergency department following a fall due to acute-onset diplopia. He reported a one-day history of shortness of breath, two weeks of purulent cough and rhinorrhea, and three days of diarrhea and vomiting.

On admission, vital signs showed blood pressure of 160/119 mmHg, pulse rate of 76 beats/minute, respiratory rate of 16 breaths/minute, and oxygen saturation of 86% on room air, which improved to 100% with supplemental oxygen. There were bilateral lower-zone lung crepitations, normal abdominal findings, and no pedal edema. Examination of the eyes showed limited bilateral adduction and supraduction with normal pupillary reflexes, suggestive of bilateral pupil-sparing third nerve palsy. He had normal power, sensation, balance, coordination, and gait. Bilateral knee and ankle reflexes were normal.

**Figure 1:**
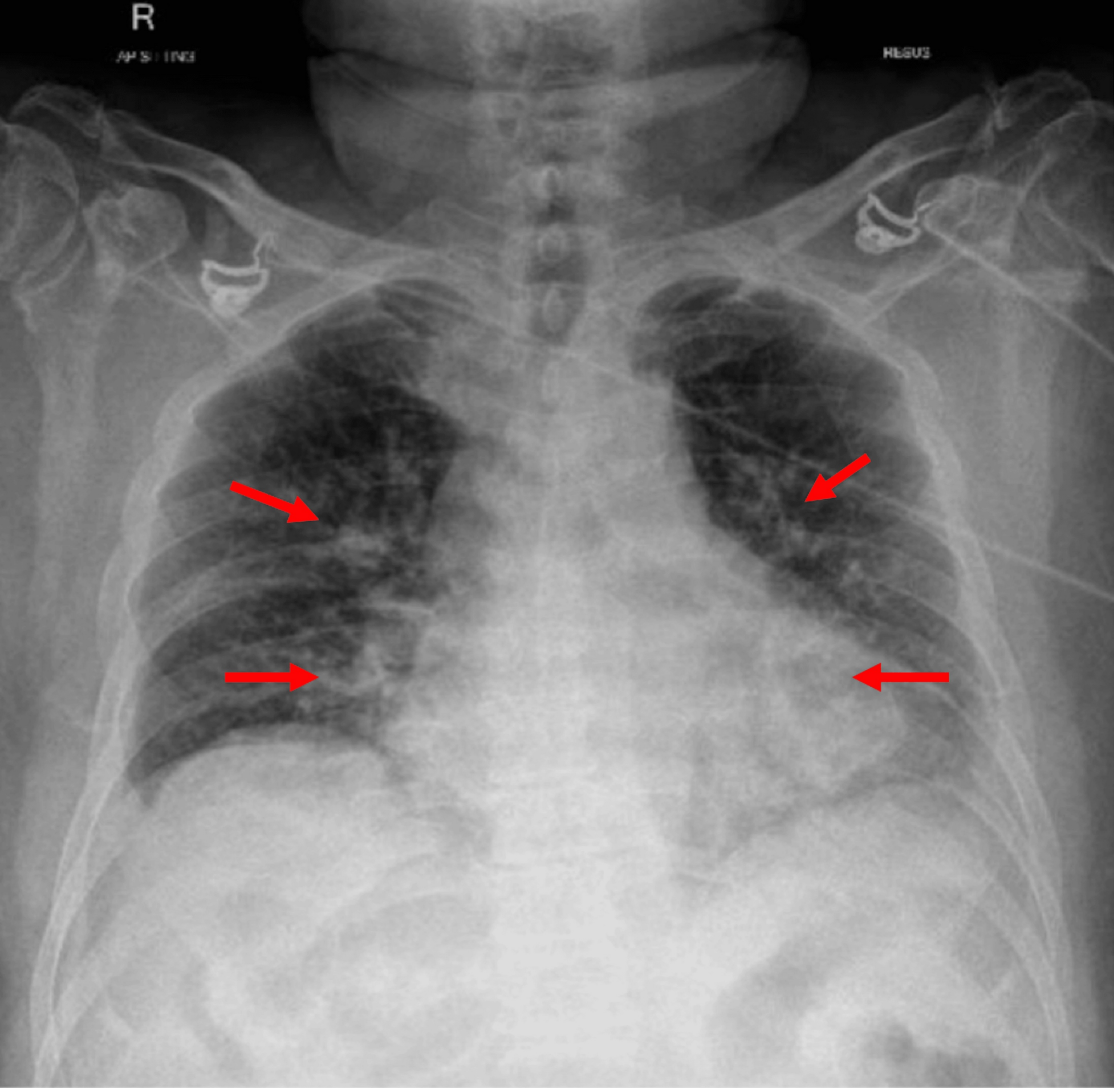
Chest radiograph showing bilateral pneumonia, with arrows indicating patchy opacities in the bilateral lower zones and perihilar regions.

Laboratory tests revealed severe hyponatremia (112 mmol/L), normal renal function, and elevated serum lactate (2.8 mmol/L). Chest radiography showed patchy airspace opacities in bilateral lower zones and perihilar regions consistent with pneumonia (Figure 1).

Initial investigations are shown in Table 1. Cerebrospinal fluid analysis was not done as the patient declined a lumbar puncture.

**Table 1:** Initial investigations.

Investigations		Value	Normal values
Complete blood count	Hemoglobin (g/dL)	15.3	12.0–16.0
	White blood cell count (×10^9^/L)	13.77	4.00–10.00
	Platelets (×10^9^/L)	305	140–440
	Neutrophil absolute (×10^9^/L)	9.92	2.00–7.50
Renal panel	Urea (mmol/L)	3.5	2.5–7.8
	Sodium (mmol/L)	112	136–145
	Potassium (mmol/L)	4.3	3.5–5.1
	Chloride (mmol/L)	80	98–107
	Bicarbonate (mmol/L)	18.2	22–29
	Creatinine (µmol/L)	57	45–84
Other investigations	Protein total, serum (g/L)	73	68–85
	Albumin, serum (g/L)	43	35–50
	Bilirubin, total (µmol/L)	27	≤21
	Alkaline phosphatase, serum (U/L)	63	40–129
	Alanine transaminase, serum (U/L)	18	≤50
	Aspartate transaminase, serum (U/L)	21.6	11.9–21.6
	C-reactive protein, serum (mg/L)	17.7	0.2–9.1

He was treated for community-acquired pneumonia with IV ceftazidime and levofloxacin, which were later switched to oral Augmentin for a total duration of 10 days. Serum C-reactive protein levels downtrended quickly from 17.7 mg/L to 4.4 mg/L by the fifth day of antibiotic treatment.

MRI of the brain was normal. Anti-GQ1b ganglioside IgG antibodies were strongly positive, confirming MFS, while acetylcholine receptor antibodies were negative. Intravenous immunoglobulin was not administered due to ongoing improvement in symptoms.

Hyponatremia was initially thought to be due to hypovolemia from gastrointestinal losses. As the patient was symptomatic, he was given a 100 mL bolus of 3% NaCl, 1,250 mL bolus of Hartmann’s solution, and subsequently started on 1 L of Hartmann’s solution over 24 hours. After an initial response, serum sodium levels dropped again on day three of admission while he was on fluid replacement. By then, the patient was euvolemic and work-up demonstrated SIADH, as illustrated in Table 2.

**Table 2:** Work-up demonstrating syndrome of inappropriate secretion of antidiuretic hormone.

Investigation	Value	Normal values
Serum sodium (mmol/L)	113	136–145
Serum osmolality (mOsm/kg H_2_O)	247	275–301
Urine osmolality (mOsm/kg H_2_O)	720	50–1,200
Urine sodium (mmol/L)	131	-
Serum cortisol (8 AM) (nmol/L)	488	133–537
Thyroid-stimulating hormone, serum (mIU/L)	1.780	0.270–4.200
Thyroxine (T4), free, serum (pmol/L)	21.6	11.9–21.6

**Figure 2:**
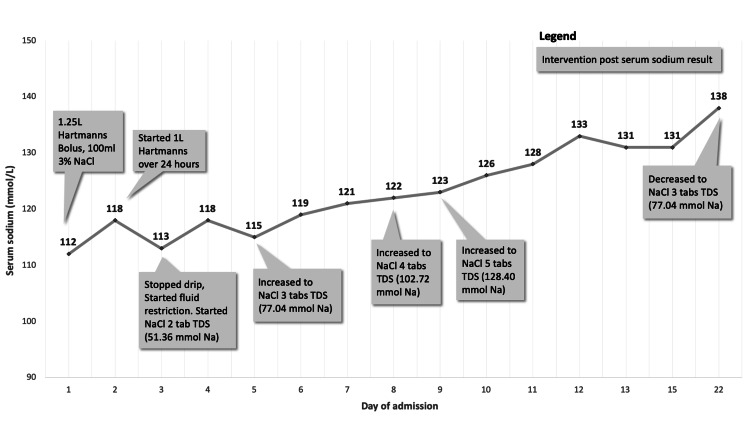
Graph illustrating serum sodium levels and corresponding treatment.

He required high-dose oral sodium chloride tablets due to persistent SIADH. Figure 2 shows a graph of the serum sodium levels during admission and the corresponding treatment.

SIADH eventually resolved eight weeks after the initial presentation, concurrently with the resolution of bilateral third nerve palsy.

## Discussion

This case illustrates the pertinent and lesser-known aspects of MFS. First, MFS can present in incomplete forms, which pose a diagnostic challenge. Additionally, SIADH can be a significant but rare complication of MFS.

Diagnostic challenges of incomplete forms of MFS

MFS is rare, with an estimated incidence of 1-2 cases per 1,000,000 people [5], and incomplete forms of MFS are underrecognized in clinical practice. Incomplete forms of MFS include acute ophthalmoplegia without ataxia and acute ataxic neuropathy without ophthalmoplegia [6]. Our patient’s presentation with isolated bilateral pupil-sparing third nerve palsy, without ataxia or areflexia, exemplifies this diagnostic challenge. Important differentials to consider for unexplained diplopia are ocular myasthenia gravis and brainstem stroke.

Retrospectively, the acuity of symptoms, presence of antecedent respiratory illness [7], and positive anti-GQ1b IgG antibodies clarify the diagnosis of incomplete MFS. Serum anti-GQ1b IgG antibodies are useful in diagnosing MFS given their high specificity and sensitivity [8]. Clinicians should thus remain vigilant to incomplete presentations of MFS and consider anti-GQ1b antibody testing early, especially in cases with ophthalmoplegia following recent infection.

Association between MFS and SIADH

Only a few cases of MFS complicated by SIADH have been documented in the literature [9-11]. The pathophysiology is presumably similar to GBS, involving autoimmune-mediated dysfunction of autonomic fibers or central regulation pathways of antidiuretic hormone [12-14].

In our patient, hypothyroidism and adrenal insufficiency were first excluded in the evaluation of euvolemic hyponatremia before a diagnosis of SIADH was made. Other common causes of SIADH were reasonably excluded, as there were no central nervous system lesions or causative medications. Although the patient had pneumonia, he promptly recovered from the infection, as evidenced by the normalization of serum C-reactive protein levels by day five of admission. The persistence of severe hyponatremia despite the resolution of pneumonia suggests that MFS is the cause of SIADH instead. Furthermore, the temporal correlation between neurological symptom resolution and normalization of sodium levels supports the relationship between MFS and SIADH. Severe hyponatremia from SIADH may cause life-threatening complications [9]. It is crucial to detect and manage SIADH promptly in patients with MFS to prevent poor patient outcomes.

## Conclusions

This case underscores several key learning points. Clinicians should remain vigilant for incomplete presentations of MFS, such as isolated ophthalmoplegia. In such cases, early testing for anti-GQ1b IgG antibodies can accurately distinguish MFS from other disorders. While SIADH is uncommon in MFS, it should be actively looked for and managed due to the potential for severe complications. Prompt recognition of SIADH in patients with MFS is essential, as targeted management including fluid restriction and sodium supplementation can significantly impact patient outcomes.
